# Optimization of Catheter Ablation of Atrial Fibrillation: Insights Gained from Clinically-Derived Computer Models

**DOI:** 10.3390/ijms160510834

**Published:** 2015-05-13

**Authors:** Jichao Zhao, Sanjay R. Kharche, Brian J. Hansen, Thomas A. Csepe, Yufeng Wang, Martin K. Stiles, Vadim V. Fedorov

**Affiliations:** 1 Auckland Bioengineering Institute, University of Auckland, Auckland 1142, New Zealand; E-Mail: ywan520@aucklanduni.ac.nz; 2 College of Engineering, Mathematics and Physical Sciences, University of Exeter, Exeter EX4 4QF, UK; E-Mail: S.R.Kharche@exeter.ac.uk; 3 Institute of Cardiovascular Sciences, University of Manchester, Manchester M13 9NT, UK; 4 Department of Physiology & Cell Biology and Davis Heart & Lung Research Institute, the Ohio State University Wexner Medical Center, Columbus, OH 43210, USA; E-Mails: hansen.296@buckeyemail.osu.edu (B.J.H.); csepe.2@buckeyemail.osu.edu (T.A.C.); vadim.fedorov@osumc.edu (V.V.F.); 5 Department of Cardiology, Waikato Hospital, Hamilton 3240, New Zealand; E-Mail: Martin.Stiles@waikatodhb.health.nz

**Keywords:** cardiac arrhythmias, atrial fibrillation, catheter ablation, computer model, patient specific model, rotors, re-entry, fibrosis, pulmonary vein isolation

## Abstract

Atrial fibrillation (AF) is the most common heart rhythm disturbance, and its treatment is an increasing economic burden on the health care system. Despite recent intense clinical, experimental and basic research activity, the treatment of AF with current antiarrhythmic drugs and catheter/surgical therapies remains limited. Radiofrequency catheter ablation (RFCA) is widely used to treat patients with AF. Current clinical ablation strategies are largely based on atrial anatomy and/or substrate detected using different approaches, and they vary from one clinical center to another. The nature of clinical ablation leads to ambiguity regarding the optimal patient personalization of the therapy partly due to the fact that each empirical configuration of ablation lines made in a patient is irreversible during one ablation procedure. To investigate optimized ablation lesion line sets, *in silico* experimentation is an ideal solution. 3D computer models give us a unique advantage to plan and assess the effectiveness of different ablation strategies before and during RFCA. Reliability of *in silico* assessment is ensured by inclusion of accurate 3D atrial geometry, realistic fiber orientation, accurate fibrosis distribution and cellular kinetics; however, most of this detailed information in the current computer models is extrapolated from animal models and not from the human heart. The predictive power of computer models will increase as they are validated with human experimental and clinical data. To make the most from a computer model, one needs to develop 3D computer models based on the same functionally and structurally mapped intact human atria with high spatial resolution. The purpose of this review paper is to summarize recent developments in clinically-derived computer models and the clinical insights they provide for catheter ablation.

## 1. Introduction

Atrial fibrillation (AF), characterized by rapid or irregular electrical activity in the upper chambers of the heart, is the most common heart rhythm disturbance [1]. When AF terminates spontaneously within seven days, or is cardioverted within 48 h, it is clinically considered as paroxysmal AF (PAF). AF is considered persistent (PeAF) when it is present for >7 days or cardioversion beyond 48 h is required to restore sinus rhythm. If a patient has AF with either unsuccessful cardioversion or AF episodes, which continue for >1 year, then the AF is categorized as long-standing persistent AF. Permanent AF is declared when attempts to restore sinus rhythm are abandoned. AF often occurs in conjunction with well-recognized heart disorders, including hypertension, valvular disease and heart failure (HF), and its incidence increases with age [2]. AF itself is not life threatening, but it increases the incidence of stroke and exacerbates heart failure [2]. About one in five stroke incidents in patients aged over 60 years are caused by AF [3]. AF affects 1% to 2% of the general population today, and its prevalence is projected to double by 2050, due to an ever-ageing population, changes in demographics, and increased incidence of HF [4].

**Figure 1:**
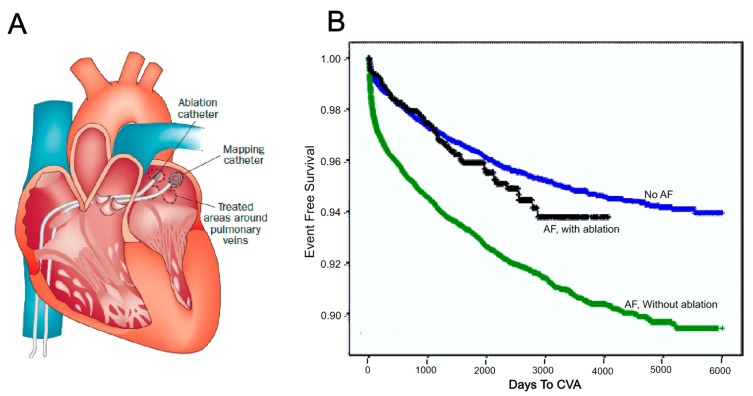
Catheter ablation and its benefits. (**A**) Ablation and mapping catheters were deployed in the left atrium for pulmonary vein (PV) isolation [9]. Reprinted from [9] with permission from Nature Publishing Group; (**B**) Long-term cerebrovascular accident (CVA) free in days for atrial fibrillation (AF) patients with and without ablation compared with those who have no history of AF [8]. Reprinted from [8] with permission from John Wiley and Sons.

Treatment of AF is an increasing economic burden in health care [5]. Generally speaking, there are three types of treatments for AF: (1) anti-arrhythmic drugs for rate control and rhythm control [6]; (2) electrical cardioversion; and (3) catheter/surgical (maze procedure) ablation. Despite recent intense clinical, experimental and basic science research activities, the success of AF treatment with current antiarrhythmic drugs and catheter therapies remains limited [7]. Radiofrequency catheter ablation (RFCA) is a widely chosen clinical option for AF treatment in patients who do not respond to antiarrhythmic drugs [5,8] and has achieved a certain level of success (Figure 1). As illustrated by Bunch and colleagues [8], AF ablation significantly lowers the risk of death in comparison with AF patients who do not receive ablation. It also lowers the incidence of stroke and dementia.

RFCA of AF aims at creating lines of block to interrupt arrhythmic electrical conduction and to prevent the AF re-entrant process [7]. The most common AF ablation techniques are wide-area circumferential pulmonary vein (PV) isolation, PV ostial ablation, linear ablation, complex fractionated electrogram ablation (CFAE), dominant frequency ablation and ganglionic plexi ablation [4,7]. Most of these approaches have proved to be effective for PAF with over 80% success rate, whereas results for PeAF have been a maximum of 50% success rate even with time-consuming “combined” (PV isolation and CFAE/linear method) substrate ablation approaches [1]. Narayan *et al.* have recently identified electrical rotors in patients with AF by using 64-channel basket catheters in both atria and demonstrated that it is possible to reverse AF in most of these patients with relatively limited ablation of atrial tissue near the rotor core in their Focal Impulse and Rotor Modulation (FIRM) trials [10]. It should be noted that their findings have been disputed by others using a variety of other approaches [11,12,13,14,15]. RFCA still requires large atrial tissue ablation near each AF driver [7,10]. More importantly, personalization of the optimal ablation strategy is difficult in clinic because each ablation made in a patient is irreversible [16].

Computer modeling offers a uniquely flexible way to test hypothesized ablation line patterns against standard clinical lesion line sets, including ablation patterns not generally performed in clinical settings due to uncertainty or high risk of the outcomes, in a reversible way in a patient-specific model to observe the impact of different ablation lesions on the AF termination in detail [16,17]. Furthermore, outcomes of patient-specific computer models can be directly validated by ablation outcomes of experimental or clinical studies. The purpose of this review paper is to summarize the recent developments on patient-specific computer models and their clinical insights for catheter ablation.

## 2. Generic 3D Computer Models

Before we discuss more detail about patient-specific models, we need to briefly introduce another important concept: the generic computer model, which employs a representative atrial anatomy and structure to illustrate the basic mechanisms behind arrhythmias. Patient-specific models are well-accepted generic computer models supplemented with clinically derived structural or functional data from a patient to investigate themes directly linked to clinical questions. However, it is important to note that only some of the atrial generic models will be mentioned in this review since this is so rich area that one focus review paper needs to be dedicated to this topic alone.

**Figure 2:**
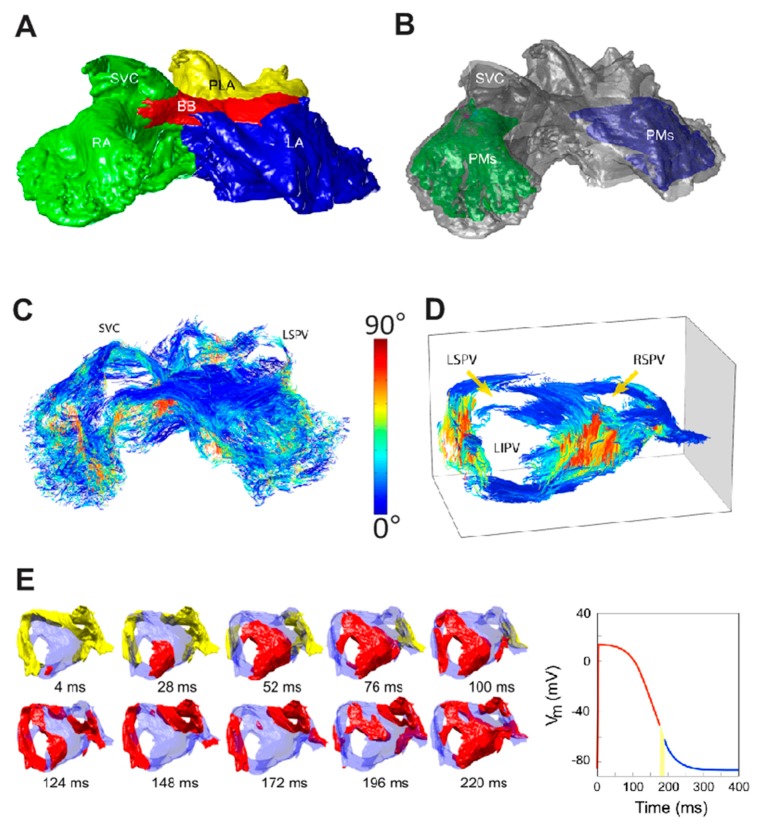
Auckland sheep atrial model [24,25]. (**A**) Epicardial surface view of sheep atria displayed from an anterior angle; (**B**) Pectinate muscle bundles (PMs) were highlighted under both atrial chambers; 3D fiber structure is displayed for the whole atria (**C**) and PLA (**D**) using a structure tensor approach; (**E**) The PLA regional computer model was employed to investigate the mechanisms behind electrical instability. SVC/IVC—Superior/inferior vena cava; BB—Bachmann’s bundle; R/LSPV—Right/left superior pulmonary veins; PLA—Posterior left atrium; R/LIPV—Right/left inferior pulmonary veins; RA/LA—Right/left atrium. Reprinted from [24,25] with permission from Wolters Kluwer Health and Institute of Electrical Electronics Engineers, respectively.

Generic computer models of atrial electrical activation provide a powerful framework for understanding the structure-function basis of reentrant atrial arrhythmias. This view has motivated numerous recent models of atrial electrical function [15,16,17,18,19,20,21,22,23,24,25,26,27,28,29,30,31], especially since Harrild and Henriquez published the first 3D electrical conduction in a realistic human bi-atrial geometry in 2000 [21]. Harrild and Henriquez’s computer model incorporates both the left and right atrial chambers and major muscle bundles, including the crista terminalis (CT), pectinate muscles (PMs) and Bachmann’s bundle (BB). This model provides the first 3D computational framework in which normal and abnormal atrial activation sequence could be closely examined. The main drawbacks of the model are that it employed stylized atrial anatomical geometry and uniform wall thickness. The second major development of 3D atrial activation models, extracted from the visible female MRI dataset, was setup by Seemann, Kharche and their co-workers [22,23]. This model utilized detailed regional segmentation of the sinoatrial node (SAN), atrial appendages, as well as regional fiber orientations of major bundles. Furthermore, this activation model takes into account electrophysiological heterogeneities across atrial chambers based on modifications of the Courtemanche *et al.* cellular model [22]. The third major atrial model was developed by the Auckland group based on the sheep atrial anatomy (Figure 2) [15,24,25,26]. In this model, 3D atrial geometry was reconstructed at a resolution of 50 × 50 × 50 µm^3^ from serial images acquired throughout the sheep atrial chambers. Myofiber orientations were then determined by the purpose-developed structure tensor analysis for the first time. These data were then incorporated into the computer model to investigate the normal and abnormal electrical activation, which emphasizes the role of atrial anatomical structure in normal and PAF sheep [24,25]. Aslanidi *et al.* have extended the structure tensor approach to the canine atria using X-ray micro-computed tomography with Iodine staining and used the resultant model to investigate the role of PVs in AF [28,29,32].

3D atrial generic models have generated rich knowledge on the basic mechanisms of atrial excitation in the past two decades:

(1) It renders us the detailed atrial anatomy for studying the anatomical features of atria, especially the anatomical bundle structure of the endocardial surface of the atria, as well as the linkage to its electrical preferential pathway during normal and abnormal conditions;
(2) It includes quantitative atrial wall thickness and myofiber architecture across atrial chambers instead of previous qualitative studies by Wang *et al.* [33];
(3) A family of atrial cellular models have been developed to fully capture atrial electrical properties under normal and diseased conditions [19].

When such biophysical detailed and anatomically accurate atrial anatomies are integrated into dynamic 3D models capable of simulating electrical excitation-propagation, they provide a powerful platform to investigate the contributing factors of atrial arrhythmias. This was demonstrated in the Auckland sheep model where heterogeneous electrical properties were assigned to the posterior left atrium (PLA) [23]. Zhao *et al.* demonstrated that vulnerability to rhythm disturbance caused by structural heterogeneity (abrupt changes in myofiber orientations and tissue volume) in the PLA is exacerbated by spatial variation of repolarization kinetics between the PVs and LA. These results provide possible explanations for the mechanisms that may trigger PAF [25].

Generic computer models provide patient-specific models with essentially all of the multi-scale methodologies from cellular activation modeling, cell-to-cell coupling, to organ level electrical propagation. Only one exception is the atrial fiber generation due to the fact that geometrical data provided through clinical imaging approaches for virtual ablation methods cannot capture myofiber structure of atria and have to rely on known rules between atrial anatomy and myofiber architecture [32,34]. Another difference between the two types of modeling is that patient-specific models are aimed to perform in clinical settings and have to be efficient or at least be able to produce long-time simulations to analyze the effects of each additional virtual ablation line on atrial electrical propagation.

## 3. Modeling Pulmonary Vein (PV) Isolations Using Clinically-Derived Computer Models

The unique advantage of 3D virtual ablation computer models is that they allow us to: (a) plan different ablation strategies on a 3D biophysical model of the atria which may include accurate 3D atrial geometry, realistic fiber orientations and detailed cellular kinetics [16]; (b) test the effectiveness of virtually infinite number of ablation configurations at a minimal cost as well as minimized ethical requirements; and (c) provide a patient-specific recommendation for the optimal ablation treatment.

**Figure 3:**
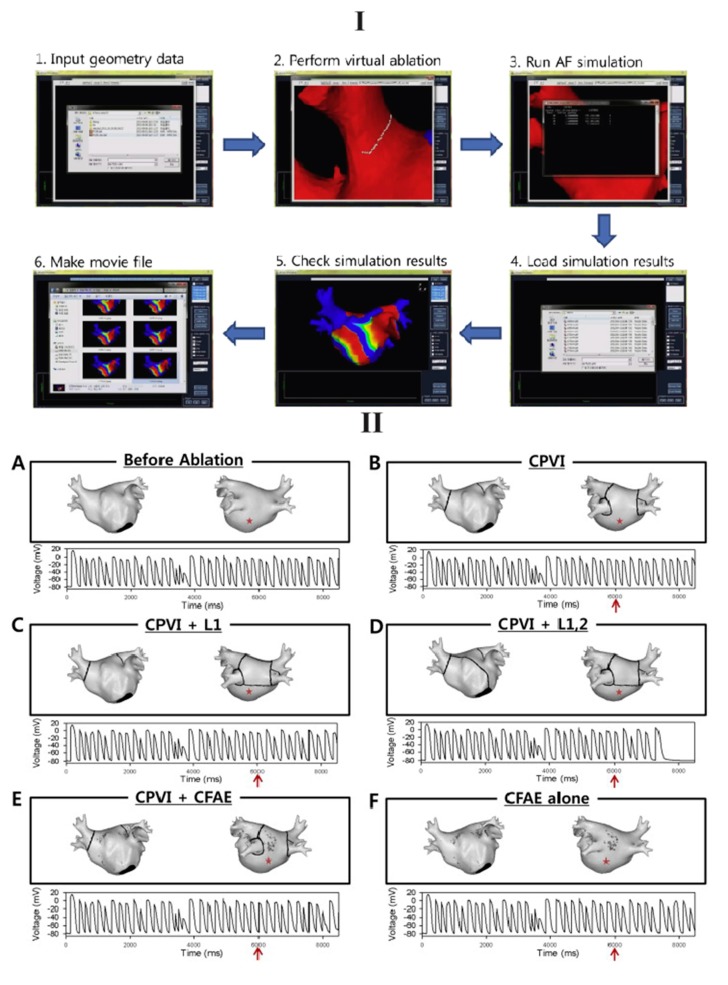
The clinical usage of a computer model for planning personalized ablation strategies was demonstrated [43]. (**I**) A GUI was displayed for performing virtual ablation; (**II**) **Top**: (**A**) pre-ablation and five ablation patterns were employed in this study: (**B**) PVI; (**C**) PVI + posterior linear ablation (L1); (**D**) PVI + both posterior and anterior linear ablation (L1,2); (**E**) PVI + CFAE; and (**F**) CFAE alone; **Bottom**: Atrial electrograms were displayed at a specified location (red asterisk) before and after (red arrow) RFCA. Reprinted from [43] with permission from Elsevier.

The first clinically-derived computer models were developed for testing a variety of PV isolation ablation strategies [35,36,37,38,39,40,41]. PV isolation is considered to be the cornerstone of RFCA for patients with PAF and is widely used as the key component of ablation lesion sets for PeAF [42]. However, whether to isolate individual PV exclusively or together, with or without additional ablation strategies, such as the linear ablation approach, is still an ongoing debate [7]. Kappenberger *et al.* have developed and studied different ablation patterns systematically using a simplified atrial geometric model of chronic AF in the past decade [20,35,36,37,39,40]. In their studies, a monolayer model instead of a structurally-detailed model was constructed based on the endocardial contour of the clinical MRI of human atria to alleviate the computational load. The database used for the ablation model contained 40 different simulated AF states and was tested with three different ablation patterns in the right atrium (RA) and six in the left atrium (LA), thus a total of 18 different combinations of ablation strategies was employed [36]. The simulation results by Ruchat *et al.* confirmed that the most complex ablation patterns led to the highest success rate and shortest time for AF termination. Uni-chamber ablation, either RA or LA, only led to success ranges of 20%–60% and 55%–80%, respectively, while bi-atrial ablation showed an increased success rate (80%–100%) [36]. More importantly, the computer model was used to obtain ablation patterns with a minimum number of lesions while reproducing the maximum success rate [35,42], *i.e.*, combining an isolation of the PVs, the left isthmus line, and the line between vena cava in the RA. In another study by the same group, a direct validation of *in silico* computer simulation results with *in vivo* data from patients who underwent RFCA demonstrated a positive correlation for AF conversion rates [37]. The most recent computer models of the LA developed by Hwang *et al.* [43] are based on clinical spiral CT scans merged with NavX geometry (St. Jude Medical Inc., Minnetonka, MN, USA) from 20 randomly selected patients who had undergone RFCA procedures to treat AF (16/20 PeAF; 4/20 PAF). In their comprehensive study, both computer simulations and empirical clinical ablation practices demonstrate that circumferential PV isolation with additional lesion lines (Figure 3D) are the most effective ablation strategy, while CFAE approach does not increase the success rate [43]. Importantly, these LA anatomical models have a constant thickness of 1.89 mm. However, atrial wall thickness variability has been shown to be an important factor in AF [44], and 3D computer models should be based on accurate atrial anatomical structure and take this variation into account. A realistic 3D human atrial model based on the visible human female data set was developed by Dossel and co-workers and then employed to evaluate 10 different PV isolation and linear line ablation strategies [41]. They concluded that the PV isolation strategy where all PVs are surrounded by continues lesion line displays the lowest success rates; furthermore, complementary linear lesions increase success rate, which is in line with clinical practices. The three PV isolation modeling studies mentioned above suggest that additional linear lesions in addition to PV isolation improves the overall success rate of RFCA, but the studies drew conflicting conclusions regarding the best approach for PV isolation. Ruchat *et al.* concluded that all PVs isolated by one circumferential lesion line yields the highest success rate, while Reumann *et al.* found that PVs isolated by one single circumferential lesion line set displays the lowest success rate. In current clinical practices, most centers employ one circumferential lesion line around PVs instead of each individual PV to avoid clinical complications and obtain higher success rate of RFCA [7]. We can see that not all outputs from computer models are reliable and useful, as correctly commented by Box and Draper, 1987, “Essentially, all models are wrong, but some are useful”.

Another outstanding clinically-derived computer model was done by Haissaguerre *et al.* [38,39]. Here, a monolayer model of AF was employed to investigate the impact of each step of the ablation procedure on PAF cycle length. First, eight locations across the atria (seven of them approximately in the PVs and one in left atrial appendage) were selected as probable representative focal pacing sources which had a wide spectrum of human atria-like frequencies from 5 to 6 Hz. As a clinically observable measurement, AF cycle length before and after each additional ablation line targeting one focal pacing source with the highest frequency was estimated. Their results demonstrated that progressive prolongation of AF cycle length was observed with the impact of source elimination. Interestingly, it displayed that at any given time, a limited number of the eight focal pacing sources with highest frequency were active, while those with lower frequency were quiescent; this observation is consistent with clinical observations [38].

Recent work by Hwang *et al.* [43] is the first study validating the outcomes of a series of individual computer models with subsequent clinical ablations, demonstrating the potential of direct clinical use of computer models at bedside and for planning personalized pre-intervention strategies. Furthermore, the authors developed a graphical user interface (GUI) for virtual AF ablation, which could be run and interpreted directly by clinicians (Figure 3) [43].

As demonstrated above, in the past decade, there have been numerous contributions from clinically-derived computer models towards a better understanding of the role of PV isolation in RFCA. These works can be divided into three categories: (1) testing the effectiveness of the performance of different PV ablation patterns, e.g., Ruchat *et al.* 2007 [36] and Reumann *et al.* 2008 [41]; (2) evaluating the impact of gradual elimination of focal pacing sources on cycle length (Haissaguer *et al.* 2007 [38]); and (3) investigating the feasibility of utilizing the virtual ablation system in clinical settings (Hwang *et al.* [43]). These computer models demonstrate that computer modeling is a powerful tool for clinical PV isolation planning and for understanding the impact of eliminating local focal sources. Importantly, these studies show that it is necessary to be aware of the limitations of computer models when designing and interpreting simulation studies.

## 4. Modeling the Role of Fibrosis in Atrial Fibrillation (AF) Initiation, Maintenance and Termination

It is well accepted that structural remodeling, specifically fibrosis, plays an important role in both PAF and PeAF [45,46]. The effect of fibrosis on atrial tissue under diseased conditions is not fully understood, but it is complex and may have multiple scales: from the membrane level (gap junction remodeling) to the cellular level (fibroblast proliferation) to the tissue level (excess collagen) [45,46]. The complex relationships among these structural factors may set the stage for AF initiation and maintenance, which is very difficult to untangle directly in the clinic. On the other hand, biophysically-detailed computer modeling provides a powerful way to dissect these complex factors and analyze their individual effects and relative contribution to AF [47]. Furthermore, the clinical data, e.g., MRI or late gadolinium-enhanced MRI (LGE-MRI), as well as associated electrophysiological recordings, which are incorporated into the computer models, will ensure the outcomes of computer simulations relate to clinical expectation.

**Figure 4:**
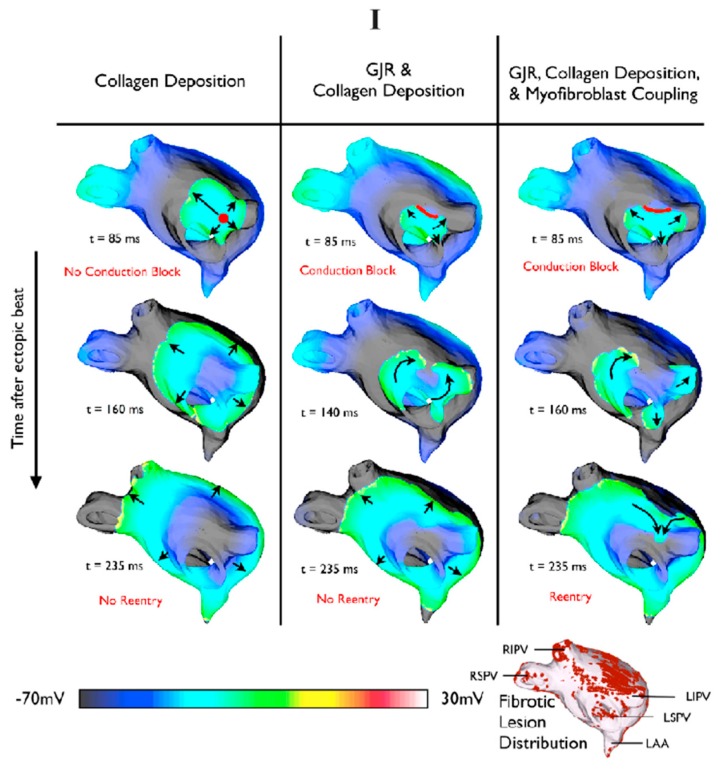

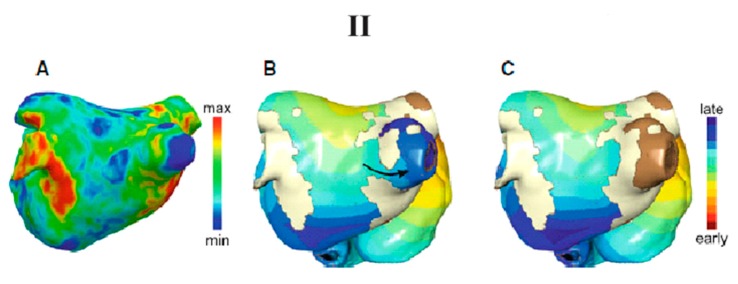
(**I**) The compound impact of structural remodeling on electrical propagations after a pulmonary vein (PV) focal beat. Reentry was observed only with both gap junction remodeling and fibroblast proliferation in fibrotic regions [53]. Reprinted from [53] with permission from Elsevier; Here the black arrows indicate the electrical propagation pathway; (**II**) A computer model of the left atrium (LA) was employed to evaluate effects of ablation lesion gaps [58]. (**A**) 3D late gadolinium-enhanced MRI (LGE-MRI) indicates the possible scars (in red) as acute results of clinical ablation; (**B**) A computer model was used to demonstrate that the incomplete ablation lines led to electrical passing through; (**C**) An additional ablation lesion was created in the computer model and tested to isolate the PV and restore the sinus rhythm effectively. The black arrow indicates the potential electrical pathway. Reprinted from [58] with permission from Springer.

The first influential computer model on the role of fibrosis in AF including both structural and electrical remodeling was done by the Michigan group [48]. The authors demonstrated that AF activation frequency, potential fractionation, and propagation dynamics are all dominated by the interaction of electrical waves with patches of fibrosis as well as anatomical structures, e.g., PV openings [48]. However, this computer model is 2D and based on a regional segment of PV sleeves. Zhao *et al.* incorporated different levels of fibrosis into the structure-detailed 3D sheep atria model and employed it to investigate the impact of fibrosis on initialization of re-entry/rotors [49]. However, they found that diffuse fibrosis has little impact on electrical propagation. Based on computed tomography (CT) imaging of RA in a patient with a history of persistent AF [50], Gonzales *et al.* went further and demonstrated that fibrotic regions could stabilize or destabilize electric rotors, and increase or decrease the rotation frequency depending their size [51]. It should be acknowledged that the most comprehensive modeling studies to date have been performed by the Trayanova’s group [52,53,54]. This group has integrated all three key elements of fibrotic remodeling into a LA model generated from LGE-MRI data of a patient with AF. In their computer simulations, AF was initiated by PV ectopic stimuli and they discovered that for structurally-remodeled human atria under the conditions of PeAF, gap junction remodeling in the fibrotic region was a necessary but not sufficient condition for the development of AF [49]. The sufficient condition as their study suggested was myofibroblast proliferation (Figure 4I). More interesting results were demonstrated in their more recent work [54], they have reconstructed four MRI-based patient-specific models of LA with increasing amount of LA fibrosis (Utah I–IV). For each model, a train of stimuli with gradually reducing coupling intervals were applied to 10 selected locations around the four PVs. They discovered that they could only induce and sustain AF in atrial models with higher level of fibrosis (Utah III and IV, >22.8%) due to slowed and discontinuous conduction, which occurred in the larger fibrotic tissue. Furthermore, they concluded for the first time that the distribution of atrial fibrosis actually dictates where the pacing sites are located in order to elicit AF, which was termed as a “sweet spot for AF induction” and demonstrated to be at intermediate distances from nearest fibrotic regions (between 0.38 and 1.05 mm). Additionally, they argued that AF is maintained by electrical rotors within confined regions, and the precise locations are decided by the distribution of fibrosis regardless of the pacing locations. These are very intriguing findings and clearly demonstrate the crucial role of fibrosis in AF initiation and maintenance. As expected, subsequent fibrosis-targeted ablation eliminated the rotors and restored sinus rhythm in the computer models [54]. However, we need to be aware that the role of myofibroblast in AF and its electrical coupling with normal myocytes is debatable [15,55]. This may be the reason for the discrepancy of fibrosis impact on electrical propagation among researchers [15,16]. Furthermore, the atrial anatomical models obtained using clinical LGE-MRI scanners lack realistic 3D myofiber orientations and high-resolution fibrosis structures [52]. As a consequence, we need to be cautious when interpreting simulation outcomes using the computer models based on low-resolution models. For example, the clinical assessment of Utah LGE-MRI fibrosis from the most recent delayed-enhancement MRI determinant of successful radiofrequency catheter ablation of atrial fibrillation (DECAAF) study concludes that overall pre-ablation fibrosis levels in a patient is positively related to the likelihood of AF reoccurrence post-ablation and decreasing the amount of residual fibrosis (the difference between pre-ablation fibrosis and ablation-induced scar) improves the arrhythmia free survival [56]. For RFCA to be effective, it is important to have transmural ablations, and any ablation lesion gaps could potentially lead to ablation failure [57]. Virtual ablation lesion sets in a computer model could help to plan the exact redo-ablation sets by localizing and testing the optimized location for additional lesions to close potential lesion gaps. A good example of this is the study conducted by Krueger *et al.*, who evaluated ablation lesion gaps in a clinical setting using an ablation computer model extracted from LGE-MRI images [58,59]. The electrophysiological simulations suggested that small gaps in the ablation lesions could reconnect PVs to the rest of LA myocardium (Figure 4II) [58,59]. Approaches such as those presented here may allow for the computer model-based evaluation of the acute and long-term success of RFCA procedures in real time at the patient’s bedside in the near future [56].

## 5. The Mechanisms of Rotors

The local source hypothesis of AF is an old concept and attributes AF to a small number of sources including reentrant rotors or focal beats [4]. Recent high-profile clinical studies by Narayan and his colleagues in FIRM trials have revealed rotors or focal drivers in nearly all patients with wide presentation of AF [10]. It has been demonstrated that AF rotors appeared in limited spatial areas for prolonged periods of time. RFCA has achieved high success rates of ablating such rotors by directly targeting their cores. However, the exact conditions that induce and permit such rotor activity in the human atria and the reasons behind the effectiveness of their targeted ablation remain unclear.

**Figure 5:**
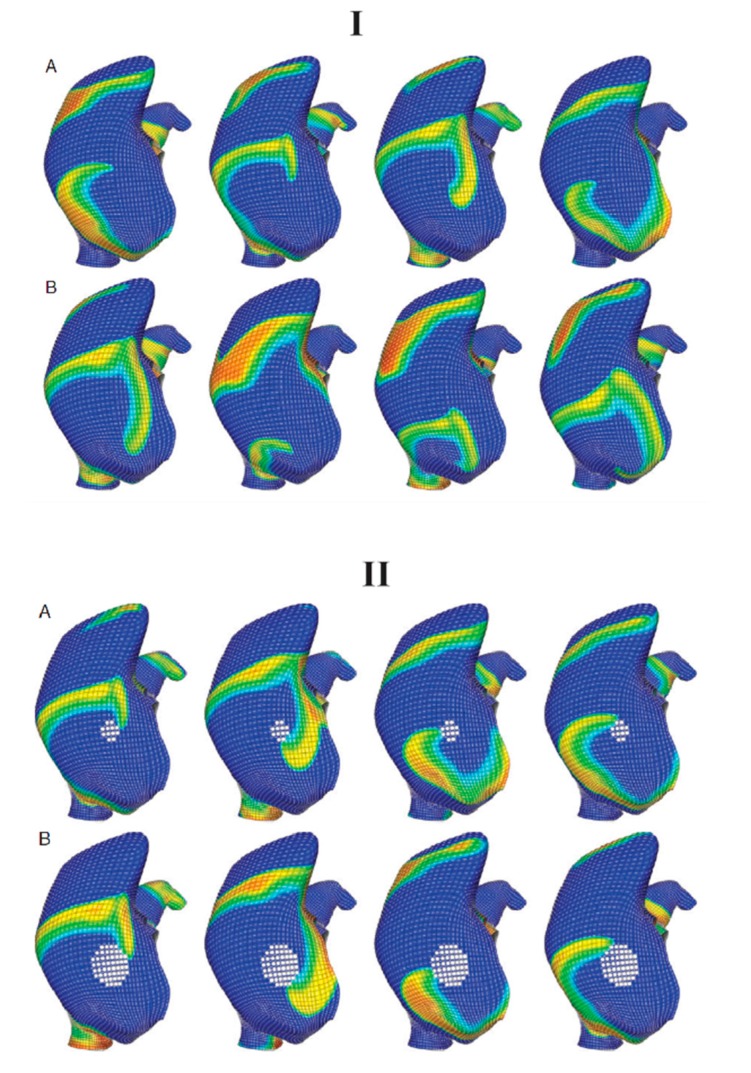
Fiber discontinuities and fibrosis were demonstrated for sustaining rotors using a computer model based on computed tomography (CT) imaging of a patient with a history of persistent AF [51]. (**I**) Fiber discontinuities (white lines) help to initialize (**A**) and stabilize a meandering rotor (**B**); (**II**) Structural remodeling, fibrosis (white area), near the rotor core converted meandering rotor to stable macro-reentry rotor. (**A**) The rotor initially meandered around the fibrotic region and eventually became sustained; (**B**) After the diameter of the fibrotic region was increased, the rotor was still stable but had reduced rotational frequency. Reprinted from [51] with permission from Oxford University Press.

The recent work by Gonzales *et al.* employed a computer model to examine the impact of atrial structural and electrical properties on rotor evolution [51] (Figure 5). This anatomical model was based on a computed tomography (CT) imaging of RA in a patient with a history of persistent AF, and this CT image was integrated with rule-based fiber orientations and regional wall thickness [50]. In their study, they discovered that reentrant rotors were readily maintained in two conditions: decreased conduction anisotropy and shorter effective refractory periods. Furthermore, they demonstrated that rotors were transiently or permanently trapped by fiber discontinuities, which is consistent with FIRM mapping studies [10]. More interestingly, their study suggested that the fibrosis region could stabilize or destabilize rotors, and increase or decrease the rotation frequency depending on the size of the fibrotic region. Potentially, this illustrates the role of fibrosis in maintaining rotors, and the reason that clinical ablation by targeting the core of stable rotors works so effectively [10,51]. Furthermore, McDowell *et al.* have demonstrated that rotor inducibility in a structurally-remodeled atria is uniquely determined by its fibrosis distribution [54]. Additionally, these rotors are confined to certain regions regardless of stimulus locations. Subsequent ablations resulting in AF termination clearly demonstrate the crucial role of rotors in sustaining AF. These two computer studies shed light on the mechanism of rotors. However, one of the key assumptions, decreased conduction anisotropy that Gonzales *et al.* took for granted in their computer model was not often observed in patients with AF, though electrical remodeling (shorter effective refractory periods) was common scenery [51]. This may lead us to doubt some of their findings. On the other hand, the simulation outcomes by McDowell *et al.* are very promising but these results need be validated by clinical/experimental studies.

Some groups have used body surface mapping to obtain potentials at the epicardial surface of the atria via the inverse method [60] and then analyze the activation patterns in patients with AF. Haissaguerre *et al.* have constructed biatrial geometry and body surface torso from CT scans of 103 consecutive patients with PeAF [12]. They discovered that re-entry rotors were not sustained (median, 2.6 rotations lasting 449 ± 89 ms, ~70% in LA and ~3% in RA), but meandered substantially and recurred repetitively in the same region. Haissaguerre’s findings are consistent with an earlier study by Rudy *et al.* [61], which claimed that the most common pattern of AF is wavelets, and that stable rotors were seen rarely, a pattern also observed by Zhao *et al.* [62]. Subsequent clinical driver ablation alone terminated 75% and 15% of PeAF and long-lasting AF, respectively [12]. However, these AF patterns obtained via the inverse solution are somewhat contradictory to the findings by Narayan *et al.* using a direct contact mapping approach [11]. Furthermore, the computer model studies by Gonzales *et al.* [51] and McDowell *et al.* [54] suggest that rotors tend to be stable and are maintained by structural discontinuity, *i.e.*, fibrosis and fiber orientation. This discrepancy may be explained by the inherent problems of inverse approach, e.g., Berenfeld *et al.* showed that band-pass filtering could reduce the apparent meandering of body surface mapped rotors (91.7% ± 5.7% *vs.* 26.9% ± 16.8%) by reducing the effect of the atrial electrical activity occurring at different frequencies [63].

## 6. Current Challenges

In the past decade, three broad categories of clinically motivated computer models have been constructed for a better understanding of AF and ablation: (1) pulmonary vein isolation; (2) atrial fibrosis; and (3) reentrant rotors. These developments reflect the dominating clinical ablation approaches at the time. There are fewer computer models developed for investigating the latter two compared with the former one since the latter two only became popular in the past five years. The computer models were being used as a quantitative tool for better understanding clinical practice. Furthermore, most of results in the past decade simply validate or better understand RFCA procedures, not to actually to guide RFCA. There are many challenges that must be overcome before computer modeling is used at a patient’s bedside in clinical settings for pre-planning and guiding RFCA [16], but here we only discuss four of them.

Firstly, current human atrial anatomical models suffer from inadequate spatial geometric resolution and are based on poor clinical MRI/CT (at most ~300 × 300 × 500 μm^3^). Most established models of the atria do not include important structural details that cause AF, such as realistic atrial wall variation, fiber orientation and fibrosis. As we know, atrial myocytes are 6 to 12 μm in width and ~80 μm in length, and some regions of the atrial wall are as thin as 1 mm [25]. Therefore, atrial 3D geometry characterized by a spatial resolution lower than 500 μm is not sufficient for computer models to utilize. It is known that the diffusion tensor MRI (DT-MRI) cannot resolve the fiber structure of the hypertrophied atria due to the extremely thin walls. In the past, only some regions of the atria were imaged using DT-MRI, e.g., the right atrial appendage of a human heart [27]. Another promising approach, called structure tensor approach, was first proposed and employed on the sheep atria with 50 × 50 × 50 μm^3^ by the Auckland group [24]. The structure tensor approach utilized the color intensity variation of the original images and 3D Eigen-analysis to model 3D fiber orientations, and has achieved great success [25]. Due to the low spatial resolution and poor color contrast of atrial image acquisition of clinical MRI/CT, the structure tensor approach can potentially yield misleading structural information [24]. Instead, a rule-based smoothed fiber pattern throughout the atrial chambers was generated and incorporated into the computer models as the most commonly used approach in patient-specific models [58]. However, regional fiber orientations from one atria to another one will be different quantitatively, though all atria share similar myofiber patterns, which is true even for different species [64]. Furthermore, myofiber databases of atria do not exist to validate the rule-based approach, and it is a challenging task to register myofiber orientations from one atria to another and generate a statistic model as it is a more established approach for ventricles [65]. Furthermore, such a rule based idealized fiber orientation may significantly alter the conclusions that the models provide. The important role of fibrosis in AF was agreed among researchers, but the only practical approach is to employ clinical LGE-MRI, which has very poor resolution with >1 mm at the *z*-axis [16]. The poor resolution of clinical LGE-MRI scanners only allows us to capture large-scale patchy fibrosis >1 mm but not small-scale patchy fibrosis and endomysial fibrosis, which are very important for AF initialization and maintenance [14]. Therefore, there is an urgent need to acquire the human atrial structure with higher resolution and with the complete underlying structure (fiber orientations and fibrosis) in order to better understand the relationship between ablation lesion sets and substrate.

Secondly, current atrial cellular activation models are far from fully capturing the realistic propagation pattern and different ionic behaviors after long pacing as demonstrated in the recent benchmarking paper by Seemann and his colleagues [19]. Therefore, we need to be cautious when we integrate these cellular kinetics into a 3D propagation model and interpret the simulation output. Ideally, we need associated functional data (electrophysiological mapping) from each patient to fully validate cellular ionic dynamics generated from computer models. To our knowledge, it has not been done in any patient-specific models yet.

Thirdly, the structurally and biophysically detailed computer models should be efficient enough to simulate long periods (in the order of a minute) of electrical propagations in order to test the impact of mimicked ablation lesions. Existing numerical solvers in the literature have drawbacks including poor parallelization, incorrect boundary conditions, and sub-optimal formulations of the biophysical problem of ablation. A robust parallel solver of the governing cardiac equations is therefore certainly warranted [24,66], such as the ongoing OpenCMISS package [67] or a novel network approach [68]. Currently, clinical models either use simplified atrial geometry/uni-chamber approach, or simple cellular activation models to alleviate the situation [16]. Therefore, an efficient version of a 3D computer model that incorporates biophysical functional and anatomical structural detail is clinically relevant [66].

Finally, and most importantly, the simulated results from computer models need to be related to clinical patient data in order to provide useful clinical insights. For this purpose, computer models must be rigorously validated by experimental or clinical outcomes before being used in predictive assessments [69].

## 7. Future Direction and Conclusions

The scientific developments discussed in this review clearly illustrate that computer modeling is a powerful vehicle for diagnosis, planning, evaluation and guidance in the clinically relevant area of AF ablation. The most powerful strength of the computer modeling approach over the clinical/experimental settings is the ability to assess the effectiveness of an unlimited number of ablation patterns in a fully controlled situation. By examining the different effects of varied ablation lesion line sets, one can optimize the ablation procedure. The recent high-profile FIRM clinical study on rotor-targeted RFCA by Narayan *et al.* [10] has achieved a much higher success rate than others and is internationally influential, potentially due to their robust mechanism-based (rotors) ablation method compared to previous empirically or anatomically based approaches [7,69]. However, it is still unclear why the FIRM approach is so effective compared with other approaches, and whether it is the optimized approach. These questions are almost impossible to tackle directly in the clinical environment, and computer models may play an important role in unraveling these uncertainties behind the RFCA.

Despite extensive study over the past decade on (1) PV isolation; (2) atrial fibrosis; and (3) reentrant rotors using clinically-derived computer models, many important and basic questions still remain unanswered, especially for the PV isolation and linear approaches. For example, what is the optimal linear approach and what is the best way to perform ablation: fibrosis *vs.* rotor region. The comparable poor performance behind patient-specific models in the past decade may be that there is a huge knowledge gap between generic models and patient-specific models, as suggested in the Section of challenges faced above. The structural and functional behaviors of human atria must be fully understood before patient-specific models are employed, since all generic atrial models are based on either animal studies or low resolution human atrial structure models which are potentially misleading. Ideally, 3D computer models of functionally and structurally mapped intact human atria, e.g., ex-planted human hearts, must be developed [70]. This will permit the accurate representation of electrical and structural properties of human atria with fine detail. Furthermore, experimental ablation can be performed on the same human heart for better validation of the computer models [71]. An optimal ablation strategy may be investigated and achieved only if patient-specific ablation lesions, which take into account different underlying atrial substrates, are employed for individuals accordingly. In computer simulations, these virtual ablation lines could be mimicked as non-conducting regions and activation frequency will be monitored and employed as an indication whether the additional ablation line works or not. Therefore, computer modeling can be used not only for pre-ablation planning, but also for guiding RFCA procedure in real-time.

The goal of such clinically-derived computer models as discussed here is to overcome critical barriers for optimal treatment of AF by developing a novel computational framework from which we can develop effective targeted treatment for AF patients. The findings of these computer simulations may change current clinical ablation practices worldwide.
